# Intergenerational Social Mobility and Youth Well-Being in the Context of the Greek Socio-Economic Crisis

**DOI:** 10.1007/s11205-022-03016-2

**Published:** 2022-10-22

**Authors:** Sophie Leontopoulou, Michael Chletsos

**Affiliations:** 1 Department of Primary Education, University of Ioannina, Ioannina, Greece; 2 Department of Economics, University of Piraeus, Piraeus, Greece

**Keywords:** Intergenerational mobility, Education, Well-being, Parental involvement, Greek crisis, Emerging adulthood

## Abstract

Intergenerational social mobility and its associations with youth well-being has scarcely been examined in Greece. This study examines educational and income mobility across generations, its relations with emerging adults’ well-being, and the impact of interpersonal and contextual factors on this relationship, such as parental school involvement and the recent severe socio-economic crisis in Greece. 468 female and male University students and graduates completed a questionnaire battery, using two different modes: pen-and-pencil and online. The questionnaire assessed demographics, including information about educational levels for youths, parents and grandparents of both genders, and income (for the former two), flourishing and quality of life, as well as parental involvement, and the impact of the crisis on youths. The results revealed upward educational mobility across three generations. Intergenerational mobility was significantly, and positively associated with youth well-being. Parental involvement mediated the relation between social mobility and flourishing, while the impact of the socio-economic crisis mediated the relationship among mobility, flourishing, and quality of life in emerging adulthood. The implications of these findings on educational, mental health and other types of interventions are discussed. Lastly, the two modes of questionnaire assessment were found to be equivalent, a finding that can facilitate research in other trying times, such as health pandemics.

## Introduction

Socio-economic inequality plunders societies the world over. It not only strips populations from their right to fairness, it also hinders their advancement at the social, psychological, economic, and institutional levels. “Patterns of inequality are imprinted from one generation to the next” (HM Government, 2011, p. 3). Intergenerational social mobility (ISM) refers to the shift in one’s social status from one generation to another (Social mobility, 2022). It is usually measured in terms of change in educational achievement, occupational and economic advancement (Bjørnskov et al., 2013; Black & Devereux, 2010). The change can be upward or downward. Such societal change causes the generation to adopt a new way of living, thinking and behaving. Differences in the parents’ and their children’s upbringing, changes in population, and changes in occupation all impact intergenerational social mobility. There is now evidence that ISM can impact well-being in different ways. Nikolaev and Burns (2014) found that socio-economic mobility can increase subjective well-being across the life span through a number of channels, including education, income and health care. They found that upward mobility is associated with positive outcomes in subjective well-being, whereas downward mobility negatively affects self-reported levels of happiness and subjective health, the effects of the latter being much stronger. Bjørnskov et al. (2013) suggested that low upward mobility strongly and negatively affects subjective well-being. Far less clear are the pathways through which ISM can exert its influence on well-being in different age groups, especially during challenging periods, such as socio-economic or health crises. The present study attempts to address certain aspects of such interrelated socio-psychological phenomena focusing on their importance for well-being in emerging adulthood. More specifically, it seeks to examine in tandem for the first time ISM and its associations with the well-being of Greek University students and graduates. It concentrates on educational and income mobility in adjacent generations. Further, it takes into account the effects of interpersonal and contextual factors, i.e. parental school involvement, and the deep socio-economic crisis that affected the country from 2008 and for at least ten years.

The Introduction is organized into three interconnected sections that review and synthesize the relevant strands in the literature. It begins with discussions of intergenerational social mobility, focusing on educational and income mobility. It moves on to explore the relationship between ISM and interpersonal aspects of well-being in emerging adulthood, zooming in on parental school involvement. It subsequently argues for the role of contextual influences on the association between ISM and well-being, highlighting the effects of the Greek crisis on University students and graduates. Throughout the text contextualized discussions are provided updating the literature on the above three themes drawing on the international and Greek experience. In addition, the epistemological and methodological foundations of the study are laid, its main terms are operationalized, and the theories that informed this interdisciplinary study are briefly stated. Finally, its main aims and research questions are presented, along with the unique lacuna that it covers.

### Intergenerational Social Mobility in Greece and Abroad: Conceptualization and Measurement

Defining ISM is challenging at best, since the concept of social change, which lies at its heart largely depends on the wider socio-economic and cultural context, as well as on other situational characteristics met in different countries. Socio-economic status (SES) has been used to index social change. It connotes one’s position in the social structure, measured usually by education and occupation, and sometimes income (Hoff et al., 2002). Economists, sociologists, and psychologists have long been interested in markers of ISM and, increasingly, on the mechanisms that underlie the relations between parents’ and children’s outcomes (Black & Devereux, 2010). Education and occupation, as well as social class and earnings have served as markers of ISM. Education has been extensively examined for its significance as a mechanism for upward social mobility, since affluent parents invest more in human capital and education for their children (Solon, 2004). Study of intergenerational transmission of education was often preferred over estimation of lifetime earnings in different generations as it is simpler to measure. As a result, there is now a wealth of literature showing that higher education is associated with higher earnings, better health, and longer lifespans, although causation and underlying mechanisms are yet to be determined.

The economic theory of the distribution of income is considered as the main theoretical context which analyses ISM (Becker et al., 2018). Distribution of income explains income inequality between different generations and between families within the same generation. ISM is the expression of inequality in income between different generations, as Becker and Tomes (1979) suggest. The income of children depends on the human and the nonhuman capital they receive, and their “endowment” which is determined by skills, ability, knowledge and other characteristics given by their family. The explanation of changes in ISM is based on the “human capital” theory developed by Becker (1964). According to this theory parents maximize their utility depending on the optimal combination between their own consumption patterns and the qualities of their children. Given the family income any increase of spending on one of two “goods” decreases spending on the other good. Parents invest in the human and non-human capital of their children, while the rate of return on human capital is more sensitive to endowments and other personal characteristics of both parents and children. The endowments of children are more closely related to the endowments of their parents. This linear relationship between parental and children income is also supported by Chetty et al. (2014b). The role of the endowments and the quantity of human and non-human capital that children receive from their parents affects their income mobility, as also mentioned by Chetty et al. (2014a), who report that the “birth lottery” is more important today than in the past as far as ISM is concerned.

ISM in Greece is difficult to determine, as structural characteristics of the Greek society confound its measurement, including its fragmented society, unregulated informal sector, flexibility without job security and social benefits, and tentative relations between education and income progression (Gialis & Leontidou, 2016). Measurement of SES is also somewhat vague, confounded by still more fluid characteristics of the country. For instance, occupational prestige does not always coincide with high financial earnings; finding a suitable occupation does not always depend on merit, but one can be pursued on the basis of social connections; and authority relations within the work environment can be influenced by fluid or established power structures. The following section focuses on the relations between education and income mobility in the country from an intergenerational point of view.

#### Intergenerational Aspects of Education and Income Mobility in Greece

In Greece a handful of studies offer insights into the role of education in ISM. Assisting children to attain the highest possible education represents not only a deep-rooted value in Greek society across generations, but also a means to higher socio-economic status, linked to higher earnings and other returns (Saiti & Prokopiadou, 2008). As Tsakloglou and Antoninis (1999) mention, in a country where education is free of charge and private education is limited, intergenerational transfers in education strongly contribute to a decline in inequality. Nevertheless, parental aspirations for their children’s educational and occupational prospects in Greece are confounded by socio-economic considerations (Vryonides & Gouvias, 2012). Parental social, cultural, and economic capital lead to different strategies, choices, and practices that affect their children’s educational attainment, producing and reproducing socio-economic differentiation in education (ibid). Moreover, although education in Greece is free, there are certain hidden costs involved. For instance, despite the fact that there are no tuition fees in Universities, families spend significant amounts even during the secondary phase of their children’s education in extra private classes. This widespread practice, known as ‘shadow education’ is thought to improve children’s prospects to enter University, but also helps maintain the status quo in educational and social mobility, as it is heavily dependent on the family’s ability to cope with the costs involved (Tsiplakides, 2018). Hence, whereas families from higher socio-economic backgrounds are able to dedicate material resources throughout their children’s school years to increase their prospects to enter University, families from lower socio-economic backgrounds struggle to afford their children similar opportunities. Therefore, socio-economic differences reflected in education may still sustain overall social inequalities, especially since social mobility in Greece has been traditionally associated with educational achievement, rather than income mobility. From the 1970s onwards secondary education was universalized and higher education significantly expanded in the country, resulting in an abundance of highly qualified degree-holders who eventually only managed to find not very demanding and adequately paid occupation (ibid). Other general characteristics of the Greek economy, such as extensive “black market’ methods to increase income also impact educational and social mobility.

In studies of educational mobility in Greece, educational movements across generations were found to take place, depending on parental characteristics, such as educational level and gender. The higher the parents’ educational level, the higher the probabilities of higher educational attainment for their offspring (Symeonaki & Stamatopoulou, 2014). In yet another study substantial educational mobility in the country was reported over the last 30 years (Daouli et al., 2010). Gender differences were unearthed to the effect that maternal education still largely influences their daughters’ educational attainment. Since parental income may affect children’s access to higher education, observed cross-generational life-time income inequalities may be attributed to the fact that the children of more affluent parents are over-represented in high-cost faculties, such as medicine or engineering (Tsakloglou & Antoninis, 1999). In a different study, paternal occupation statistically significantly affected adult poverty levels (Papanastasiou & Papatheodorou, 2018). This effect prevailed among the extremes on the occupational ladder, with a particularly negative effect for people from lower socio-economic families. The following section zooms into the relations between ISM and youth well-being.

### Intergenerational Social Mobility and Youth Well-Being: Interpersonal Influences

The links between ISM and well-being have long been studied (Becker & Birkelbach, 2018; Bjørnskov et al., 2013). Well-being has been empirically treated both as a predictor and an outcome marker of social mobility, defined as social, economic, and educational status. Upward and downward trajectories of ISM have been found to impact well-being; the evidence, however, is not equivocal. Some researchers maintained that both upward and downward social mobility affect socio-economic advancement and well-being for different reasons each (Black & Devereux, 2010). Drawing data from a longitudinal UK study Li (2016) explored how social class affects social connectedness and perceived quality of life through intergenerational social mobility. Perceived health status, happiness and overall life satisfaction served as indicators of well-being. Social class was found to exert direct and indirect influences on well-being. In addition, social capital, treated as resources residing in formal and informal social networks (i.e. civic engagement, neighborhood cohesion, network diversity, and network size) had a significant, albeit small impact on well-being.

Family processes including parenting behaviors have been often linked to good developmental outcomes, such as academic achievement and well-being in childhood, adolescence, and later in life. Longitudinal data from the US suggested that children’s perceptions of parental school involvement and emotional involvement influence their well-being in adolescence (Wenk et al., 1994). Other studies reported that academic and social self-efficacy mediated the relationship between paternal and maternal involvement and subjective well-being in adolescence (Yap & Baharudin, 2016). Parental school involvement, alongside processes such as parental socialization, behaviors and expectations and other family processes has been repeatedly found to influence the way economic status affects positive youth outcomes (Shanks et al., 2010). For instance, financial strain can negatively affect adolescent academic achievement through decreased parental school involvement and negative parent-adolescent relationships (Gutman & Eccles, 1999). On the other hand, increased levels of parental involvement can lead children of more socially advantaged parents to privileged track placements in high schools, thus facilitating their chances of entering college (Lucas, 2001). With regards to the relations between ISM and parental behaviors, a recent research project reported that in rural China parents desiring educational success for their children tended to be more involved  in their children’s schools, as a means of gaining social mobility (Kong, 2015).

The impact of education and income mobility on the well-being of young students and graduates in Greece is far from clear. Not only the role of interpersonal factors, such as parental school involvement has not been examined for Greek youths, other contextual factors further complicate its measurement. The recent pervasive socio-economic crisis (2008–2018) clearly affected the way young University students and graduates navigated the challenges, normative and non-normative, associated with emerging adulthood, as well as their influence on their well-being and mobility. The following section examines the impact of crises on the relationship between youth well-being and intergenerational mobility.

### Contextual Influences on Intergenerational Social Mobility and Well-Being: The Impact of the Greek Economic Crisis on Young People

To the extent that social phenomena co-exist and influence each other, economic crises arguably exacerbate pre-existing adverse socio-economic situations to impact economic and psychological well-being at a national level. They also affect the young more decisively than other age groups for a number of reasons. Emerging adults are more economically vulnerable than older generations, as they have not necessarily entered the job market, and are, therefore, exposed to increased insecurity regarding their prospects of finding and maintaining a job with adequate wages, satisfying working conditions and potential for job advancement (Kretsos, 2014; Leontopoulou, 2018). The crisis of the Greek economy in 2008 affected income inequality, as well as poverty levels, income and wage mobility. According to Andriopoulou et al. (2018) there was a decrease of the household income by more than 40% and the unemployment rate stood at 27%. Unemployment affected the younger employees more than the older ones, and younger people lost a greater part of their income than older people (Matsagganis & Karakitsos, 2020). The influence of economic crises may be more detrimental to the pursuit of positive youth development. Studies that have been carried out since the onset of the Greek crisis in 2008 reported that emerging adults lacked personal goals, had high rates of pessimism, reduced self-confidence, and poorer academic outcomes (Frangos et al., 2012), as well as a sense of abandonment and a loss of confidence in state institutions. In a study of the impact of the Greek crisis on adolescents and their families, as manifested by increased parental unemployment, tensions in the family and lowered ability for holidays and private tuition, was largely negative: students’ life satisfaction dropped, while cannabis use increased, although smoking and alcohol consumption decreased (Kokkevi et al., 2014). Motti-Stefanidi and Asendorpf (2017) in a study of adolescents before and after the emergence of the crisis also reported largely negative symptoms in youths, such as worse behavior in school, high absenteeism and lower self-efficacy; however, their overall levels of well-being did not appear to be affected post crisis, while some even reported higher academic achievement. Further, a set of four studies at the beginning of the crisis also suggested that socio-economic status may mitigate the effects of the crisis (Leontopoulou, 2018). Youths from families of a higher socio-economic status reported better psychosocial outcomes, such as higher levels of self-efficacy, emotional intelligence, sibling and parental support, school engagement and resilience, as well as mental health and more positive perceptions of hypothetical and actual negative events.

Despite evidence of some positive results amidst the crisis, an unprecedented 50% youth unemployment in Greece in 2016 (Papanastasiou & Papatheodorou, 2018), coupled with the more negative sides of the crisis were some of the main factors that led to a significant “Brain Drain”, where a large proportion of the better educated, and perhaps the more promising youths of their generation sought employment abroad. Those who remained in the country mainly constitute the “young precariat”: a term used to refer to the young generation who were ready to enter the job market when the crisis hit in 2008. These young people are exposed to a set of particular politico-historical and economic conditions, such as prolonged transition to adulthood and independence, precarity, or work instability, accompanied by serious material and psychological effects on their overall quality of life (Galanaki & Sideridis, 2019). The “young precariat” seem to experience worse socio-economic conditions compared to their parent generation, while the crisis increased the levels of precarity (Gouglas, 2013). One particular form of precarity involves patterns of atypical employment, such as seasonal, part-time, temporary, solo self-employment, and family work. It expanded unevenly across Greece during the recent crisis, while new, more precarious forms of employment emerged (Gialis et al., 2017). Family-help, ‘a long-standing, socially embedded form of atypical employment in Greece (5.4% in 2011)’ (ibid, p. 6) mainly affected youths from lower-middle class. Meanwhile, other young people in Greece and abroad found themselves not in education, employment, or training, and were termed NEET. According to OECD data (2018), the prevalence of NEET status in the crisis affected Greece was 25%. In a recent study Greek NEETs compared to non-NEETs were older, originated from families with lower income, and were uninsured (Basta et al, 2019). These less-privileged youths in Greece arguably suffered poor mental health and well-being, while older and long-term NEETs appeared to experience higher anxiety and moderate depression symptoms. It is conceivable that these youths possibly perceived their prospects of upward social mobility as seriously compromised. In a different study Papadakis (2013) reported that 54.6% of young Greek NEETs experienced feelings of anxiety, 31.7% of hopelessness and 15.9% of loneliness and exclusion from society. These findings can inform the ongoing discussion of patterns of ISM and well-being for the more- and less-privileged youths in Greece and abroad.

### The Present Study

Various theoretical perspectives informed the conceptualization and design of this interdisciplinary study. The economic theory of the distribution of income (Becker et al., 2018) was used to better understand the impact of ISM on youth well-being. Positive psychology, a recent paradigm of psychological theory, research and practice offers a novel point of view focused on the positive aspects of human experience accompanied by new ways to measure well-being, the most recent being flourishing (Diener et al., 2010). This perspective was adopted here, and intrapersonal and interpersonal measures, such as flourishing and quality of life served as indicators of well-being in emerging adulthood. In line with positive psychological thinking, parental involvement, a particular type of family processes was treated as an interpersonal asset that can influence the relationship between ISM and well-being in emerging adulthood. In addition, Arnett’s (2000) developmental theory was adopted to describe how this new life period poses distinct developmental challenges for youths aged 18–29, be them normative (i.e. identity formation, exploration of possibilities), non-normative (i.e. economic or health crises), or social, relationship, economic, and vocational ones, which tax their capabilities and resources. Politico-historical and economic conditions represent generational events that can massively impact economic and psychosocial well-being (Gouglas, 2013), especially for the young. Arguably, the multifaceted crisis described previously still impedes positive developmental trajectories of Greek youths today. Its’ deep, pervasive, and lasting effects are, therefore, examined in this study for their potential to influence the relationships between ISM and well-being in emerging adults.

#### Aims and Hypotheses

The above three theoretical perspectives were combined to highlight up-to-date aspects of ISM in Greece today, and to describe, measure and explain intricately related phenomena, focusing on University students and graduates’ well-being under crisis. Thus, the present study advances the literature in several ways. For the first time in the Greek literature, but also internationally it brings together in an interdisciplinary framework different aspects of ISM and well-being. It uses multiple indicators, both well researched and novel ones to examine both concepts, namely education and income for the former, and flourishing and quality of life for the latter. The study focuses on emerging adults, an understudied group, as far as social mobility is concerned, especially in connection with the effects of an economic crisis. It employs state-of-the-art analysis (Structural Equation Modeling) to better handle a wealth of information for three consecutive generations, including their socio-economic status and parental practices that may lead to youth well-being under extreme socio-economic conditions. Hence, the paper covers a unique lacuna: it attempts to synthesize different scientific traditions, including theories and measurement derived by them. It brings emerging adults into the forefront in order to address issues largely ignored in the literature, not only in Greece, but also abroad, such as youth well-being, as related to ISM during a severe crisis.

The study has a secondary, albeit no less important methodological focus. As the use of technology becomes more widespread, the equivalence of in-class pen-and-pencil and online questionnaire completion methods needs to be established. Both modes were used in this study to explore the potential usefulness of a technology driven approach to access participants and measure their responses. This mode is now more relevant than ever globally, since the Covid-19 pandemic has driven people worldwide to study, work, connect and participate in various cultural, social and civic activities long-distance, using online technologies.

The study hypothesizes that (a) there is upward ISM in education in Greece across three generations (grandparents, parents and emerging adults), (b) ISM affects psychological well-being in emerging adulthood, (c) parental school involvement mediates the relationship between ISM and youth well-being (flourishing and quality of life), (d) the socio-economic crisis also mediates this relationship, and e) the pen-and-pencil and the online completion of questionnaires are equivalent.

## Method

### Sample

A total of 486 youths participated in the study, of whom 390 (80.2%) were female and 96 (19.8%) male. Mean age was 20.77 yrs (*SD* = 2.36 yrs). The vast majority of the sample (95.3%) were University students (89.3% undergraduates, 6% postgraduates), while 4.7% had finished their studies. 64% studied Humanities, 11.2% Finances and Management, 5.1% Science, 2.5% Health Sciences, 2.1% Social Sciences, 1.7% Polytechnic, and 13.3% other. Only 20.6% of participants were in employment, while 79.4% were not. As a proxy for the socio-economic status (SES) of participants, paternal SES was used. Thus, 34% originated from higher SES families, 27% from average, and 18.5% from lower SES families (20.6% missing values). Participants’ mean monthly income was €425.83 (*SD* = €80.13) and mean family income was €2097.3 (*SD* = €237.3). 36.6% lived on their own, 34.4% lived at home with their family, 7.7% with a partner, 6.4% with relatives, 6.2% with a friend, 5.2% at a University Hall of Residence, 2% with a sibling and the rest had other living arrangements. 73% were moderately and very satisfied with their living arrangements, 20.3% were neither satisfied not dissatisfied, and 6.7% were unsatisfied. 96.3% of the sample were single, 2.3% married and 1.4% other. 59.7% were in a romantic relationship, a further 40.3% were unattached.

Two modes of responses to the questionnaire battery were implemented, either pen-and-pencil, or online survey. 343 (70.6%) youths completed the questionnaire battery during course time at the University of Ioannina in Greece, while 143 (29.4%) responded to the online questionnaire at their own time.

### Measures

A questionnaire battery was compiled to assess the relationships between the study variables.

*Demographics* measured in the study included gender, age, student status (graduate, postgraduate, or not a student), type of study, work status, highest level of educational attainment for participant, mother, father, grandmother and grandfather, participant and parental income, living situation and satisfaction with it, marital status and romantic involvement.

*Parental involvement* was indexed using a 6-item scale, which featured the six types of parental involvement suggested by Epstein and Salinas (1993) and Carey et al. (1998), as adapted by Bonia (2011). The scale consists a 4-point Likert-Type scale (1 = Never to 4 = Often). Sample items include (How often do your parents) “Check if you have done your homework” and “Participate in parent-teacher meetings”. A high score indicates higher parental involvement. Cronbach’s alpha in this study was α = 0.73.

*Impact of the economic crisis* were measured with two custom questions developed for the purposes of this study. The first question asked participants to characterize their family’s ability to meet its basic needs, while the second to characterize their family’s overall financial situation. The questions were answered on a 5-point Likert-type scale ranging from 1 = very poor to 5 = very good. A higher score indicated fewer negative consequences as a result of the crisis. Cronbach’s alpha was α = 0.69.

The first indicator of *well-being* was *flourishing,* measured with the use of the Flourishing Scale (FS. Diener et al, 2010). Responses to the scale’s 8 items ranged from 1 = Strongly disagree to 7 = Strongly agree. Sample items include “I lead a purposeful and meaningful life” and “I am a good person and live a good life”. High scores indicate that respondents view themselves in positive terms in important areas of functioning, such as positive relationships, feelings of competence and meaning and purpose in life. Cronbach’s alpha in this study was α = 0.80.

Well-being was also measured as *quality of life,* indexed by the Quality of Life Scale (QOLS. Flanagan, 1978; Burckhardt et al., 1989). The scale consists of 16 Likert-type items ranging from 1 = Terrible to 7 = Delighted. Sample items include (how satisfied are you with…) “Health-being physically fit and vigorous” and “Close friends”. A high score indicates increased sense of quality of one’s life. Cronbach’s alpha in this study was α = 0.85.

### Procedure

Permission to distribute questionnaires to students for completion during course time was sought from academics who taught at the Humanities, Finances and Management, Science, Health Sciences, Social Sciences, Polytechnic and other academic departments at Institutes of Higher Education in Northern and Western Greece. Students signed informed consent forms prior to completing the survey. Questionnaires were administered during lectures and took about 20’ to complete. The online version of the questionnaire battery was available for youths to complete for the same period. Students who had already participated in the pen-and-pencil version of the study notified their peers, and academic instructors who administered the questionnaires during their courses also posted information about the study at their courses’ webpages. Participation in the online version of the study was restricted to emerging adults aged 18–29 years who were currently studying towards or had recently attained a higher education degree.

## Results

### Demographics

Table 1 shows means, standard deviations and Pearson correlations among well-being measures, parental involvement, and the crisis effects measures. Next, a set of descriptive analyses were performed to identify any differences among the study participants. Initially, potential differences were explored between those youths who completed the pen-and-pencil and the online version of the questionnaire. Significant differences included the following. Youths who took part in the online condition were slightly older than those who completed the questionnaires manually (t (424) = − 5.17, *p* < 0.001). More males participated in the pen-and-pencil condition than in the online condition (χ^2^ (1, N = 96) = 6.56, *p* < 0.01). More students of Humanities partook in the pen-and-pencil condition (χ^2^ (6, N = 472) = 74.28, *p* < 0.001) and more Finances and Management students in the online condition. More female Humanities students took part in the pen-and-pencil condition than in the online one (χ^2^ (6, N = 472) = 63.22, *p* < 0.001). More youths who had finished their studies and who originated from higher SES backgrounds took part in the online condition (χ^2^ (2, N = 486) = 36.62, *p* < 0.001 and χ^2^ (3, N = 486) = 19.00, *p* < 0.01 respectively). Thus, all further analyses were carried out for the whole sample. Subsequently, significant differences were identified in favor of students of Humanities relative to those taking different courses: they came from higher SES families (χ^2^ (18, N = 472) = 33.26, *p* < 0.05), did not work (χ^2^ (6, N = 471) = 16.54, *p* < 0.01), were in a romantic relationship (χ^2^ (6, N = 466) = 13.79, *p* < 0.05), and were not married (χ^2^ (26, N = 24) = 52.88, *p* < 0.001). Undergraduate students in their vast majority did not work (χ^2^ (2, N = 482) = 23.27, *p* < 0.001), and were not married (χ^2^ (8, N = 480) = 48.79, *p* < 0.001). Finally, demographic differences were identified for the main study variables with the use of a series of ANOVAs and are shown in Table 2.

**Table 1:** Means, SDs and Pearson correlations among well-being measures (quality of life and flourishing), parental involvement, and the crisis effects measures

	Mean	SD	Quality of life	Flourishing	Crisis effects
Quality of life	5.35	.74			
Flourishing	5.68	.75	.63***		
Parental involvement	4.42	11.46	− .00	− .09*	
Crisis effects	3.71	.79	.21**	.18**	− .02

*p < .05, **p < .01, ***p < .001

**Table 2:** Demographic differences (ANOVAs) for the main study variables

	Quality of life	Flourishing	Parental involvement	Crisis effects
Gender			Females > Males *F*(1, 484) = 20.80***	
Type of studies	Medicine > Humanities *F*(1, 484) = 20.80***			Medicine > Humanities *F*(6, 465) = 2.05*
Student status		Students > Graduates *F*(2, 483) = .21*		
Work	Working > Non-working *F*(1, 483) = 6.45**			
SES-Participant	Middle, Higher > Lower *F*(3, 482) = 3.78**		Higher > Lower *F*(3, 482) = 3.56**	
SES-Father			Higher > Lower *F*(3, 482) = 8.77***	Higher > Lower *F*(3, 482) = 6.44***
SES-Mother			Higher > Lower *F*(3, 482) = 9.75***	
SES-Grandfather			Higher > Lower *F*(3, 482) = 4.08**	Higher > Lower *F*(3, 482) = 2.89*

*p < .05, **p < .01, ***p < .001

### Intergenerational Mobility in Education

A set of analyses were carried out to determine the patterns of intergenerational educational mobility in the study. As far as absolute mobility rates are concerned, the total mobility rate indicated that ISM for fathers and participants was Μ_r_ = 71.76, and for grandfathers and fathers Μ_r_ = 81.25. Both indicators suggested high intergenerational mobility. Moreover, high intergenerational upward mobility was observed for fathers and participants, M_u_ = 70.91, and for grandfathers and fathers M_u_ = 70.5. Conversely, downward ISM was low, where mobility for fathers and participants was M_u_ = 0.84, and slightly higher for grandfathers and fathers M_u_ = 4.74. Moderate to low intergenerational stability rates were observed for fathers and participants, I_N_ = 28.23, and for grandfathers and fathers, I_N_ = 18.75.

With regard to relative rates of ISM Pearson r correlations between percentile ranks were estimated. Statistically significant, albeit low correlations were found between the percentile ranks of the educational level of young people and their father (r = 0.16, *p* < 0.001), and also their mother (r = 0.16, *p* < 0.001). Statistically significant and moderate correlations between percentile ranks of the educational level for fathers and grandfathers (r = 0.34, *p* < 0.001), as well as their grandmothers (r = 0.31, *p* < 0.001) were also observed. Next, the probability that a child reaches the top fifth of the distribution conditional on parental quintile was calculated. This is a relative social mobility rate that reflects patterns of social fluidity and is calculated using multinomial logistic regression. Tables 3 and 4 show results only for the top two educational levels in Greece, i.e. (former) Technological Educational Institution (TEI) and University.^1^

**Table 3:** Probability (%) that the child reaches the two higher educational levels in Greece depending on the educational level of their parents

Parental education	Participant education					
	TEI			University		
	P. R	Observed (%)	Predicted (%)	P. R	Observed (%)	Predicted (%)
*None*						
Father	.38	24.1	21.2	− .38	75.9	78.8
Mother	.24	26.1	23.9	− .24	73.9	76.1
*Lower*						
Father	− 1.02	10.2	13.8	1.02	89.8	86.2
Mother	− .78	11.3	15.2	.78	88.7	84.8
*Middle*						
Father	1.37	12.3	8.7	− 1.37	87.7	91.3
Mother	.68	10.8	9.2	− .68	89.2	90.8
*Technical*						
Father	− .53	4.1	5.3	.53	95.9	94.7
Mother	− .45	4.4	5.5	.45	95.6	94.5
*Higher*						
Father	− .15	3.0	3.2	.15	97.0	96.8
Mother	.00	3.2	3.2	.00	96.8	96.8

None = until 2nd class in Primary School, Lower = until 3rd class in High School, Middle = finished Lyceum, Technical Education = ΙΕΚ, ΚΕΚ, Technical Schools, and Higher = ΤΕΙ, University

P. R. = Pearson Residual

**Table 4:** Probability (%) that the father reaches the two higher educational levels in Greece depending on the educational level of his parents (grandparents)

Grandparent education	Paternal education					
	TEI			University		
	P. R	Observed (%)	Predicted (%)	P. R	Observed (%)	Predicted (%)
*None*						
Grandfather	4.29	5.9	0.3	− 0.14	0	0.1
Grandmother	6.17	9.09	0.2	− .08	0	0.1
*Lower*						
Grandfather	3.96	23.5	4.2	− 0.73	0	1.8
Grandmother	− 0.55	0	2.7	− .38	0	1
*Middle*						
Grandfather	.58	23.5	18	− 0.51	10	13.2
Grandmother	− 0.68	9.1	16.8	.14	13.3	12.1
*Technical*						
Grandfather	.5	29.4	24.2	− 1.13	13.3	21.9
Grandmother	1.23	45.5	28.6	.42	33.3	28.3
*Higher*						
Grandfather	− 2.94	17.6	53.3	1.54	76.7	63.1
Grandmother	− 1.01	36.4	51.7	− .4	53.3	58.5

None = until 2nd class in Primary School, Lower = until 3rd class in High School, Middle = finished Lyceum, Technical Education = ΙΕΚ, ΚΕΚ, Technical Schools, and Higher = ΤΕΙ, University

P. R. = Pearson Residual

Overall, increased ISM was observed for education between all three generations examined in the study. For instance, the probability that youths with parents from the lowest educational status reach the highest educational level in the country (i.e. University) was 75.9% for fathers and 73.9% for mothers. On the contrary, the probability that the father reached the highest educational level, when his own parents came from the lowest educational background were zero. The probability that children with parents from the highest educational background also entered University was 97% for fathers and 98.6% for mothers. A generation back, fathers with parents at the highest educational levels entered University much less frequently, i.e. 76.7% when their fathers were University educated, and 53.3% when their mothers were University educated.

#### Intergenerational Mobility and Youth Well-Being in the Face of Crisis

**Fig. 1:**
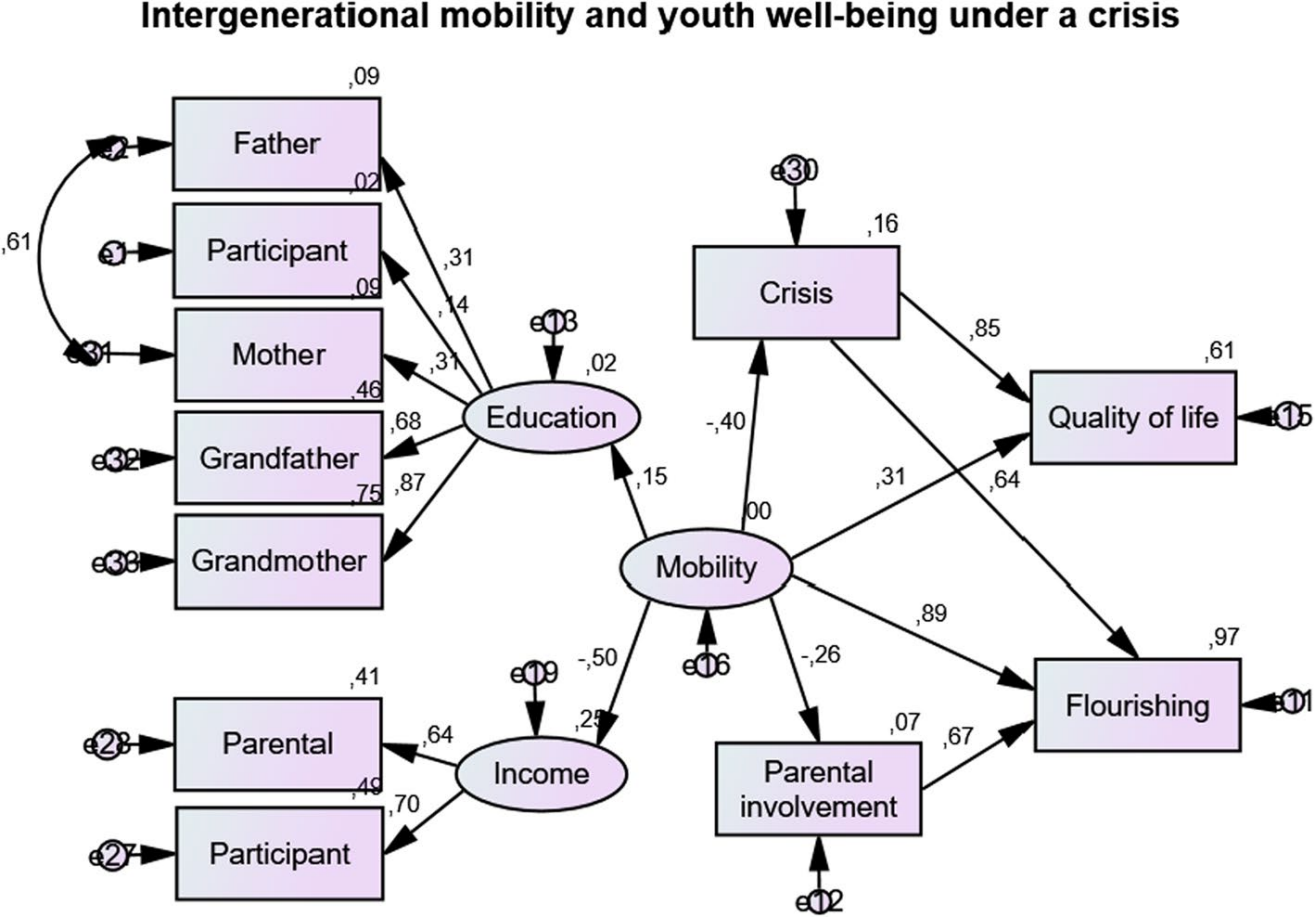
SEM model of intergenerational social mobility measures associated with youth well-being measures, with parental involvement, and a socio-economic crisis as mediators (*n* = 486)

The final step in the analysis was designed to examine the main variables of the study in their interrelations. The links among intergenerational social mobility, and the well-being of emerging adults, as influenced by parental involvement and the socio-economic crisis in Greece were explored with the aid of Structural Equation Modelling (SEM. Kline, 1998). SEM is a family of statistical procedures that combine techniques, such as path analysis, confirmatory factor analysis and regression analysis that can offer in depth understanding of theory driven research. The SEM modelling was carried out using AMOS v. 22 (Arbuckle, 2013), using maximum likelihood estimation. Figure 1 shows the standardized path coefficients of the structural equation model. ISM was measured as a latent variable comprising levels of education in three generations, i.e. participants, parents and paternal grandparents, and income in two generations, i.e. participants and parent (income of main wage earner in the family, either mother or father, as indicated by participants).

Regarding model fit, the χ^2^ is a sensitive measure, especially when sample sizes are large, or the observed variables are non-normally distributed. The χ^2^ for the causal model examined in the study was very good, χ^2^ (39) = 109.28, *p* = 0.000. The root mean square error of approximation (RMSEA), a measure of the discrepancy in fit per degrees of freedom also indicated a good fit, RMSEA = 0.06. The other indexes used here confirmed the model’s good fit: CFI = 0.94; CMIN/DF = 2.8, *p* = 0.000; NFI Delta1 = 0.92; AIC = 185.28; and PCLOSE = 0.08. The model explained 96% of the variance of flourishing, 60% of quality of life, and only 6.7% of parental involvement. Figure 1 indicates that ISM was significantly associated with youth well-being, as indexed by quality of life and flourishing in emerging adulthood. It also demonstrates that the effects of the socio-economic crisis significantly and highly mediated the effects of mobility on youth quality of life and flourishing, but not on parental involvement. Rather, parental involvement proved to mediate the relation between mobility and flourishing significantly and highly.

Overall, this model seems to adequately explain the complex relations between the study variables. ISM was the strongest direct predictor of flourishing in youths, but it also significantly predicted both quality of life and parental involvement (negatively). In addition, mobility was significantly and negatively related to the socio-economic crisis. The crisis was the strongest predictor for quality of life, while it also strongly predicted flourishing in emerging adulthood. Parental involvement, on the other hand, did not seem to relate to the crisis; rather, it was strongly associated to flourishing, both directly, and as a mediator for mobility. Table 5 shows unstandardized estimate, standard error, and standardized estimate of each indicator of the latent and predictor variables of the outcome variables for the SEM model.

**Table 5:** Unstandardized estimate, standard error, and standardized estimate of each indicator of the latent and predictor variables of the outcome variables for the SEM model

			Unstandardized estimate	S.E	*P*
Parental involvement	←	Mobility	− 149.18	86.97	.08
Education	←	Mobility	1		
Income	←	Mobility	− 33,082.58	16,867.71	.05
Crisis	←	Mobility	− 13.57	7.662	.07
Flourishing	←	Parental involvement	11.56	.04	***
Quality of life	←	Mobility	379.95	5.86	.04
Flourishing	←	Mobility	1		
Participant education	←	Education	3.29	206.29	.06
Father education	←	Education	1		
Participant income	←	Income	2.58	1.28	.01
Parental income	←	Income	.93	.37	***
Quality of life	←	Crisis	8.07	.04	***
Flourishing	←	Crisis	3.05	1.37	***
Mother education	←	Education	9.87	1.19	.01
Grandfather education	←	Education	.93	3.58	.00
Grandmother education	←	Education	7.36	2.74	.00

***p < .001

## Discussion

This interdisciplinary study set out to explore the intricate relations between ISM and the well-being of University students and graduates, as influenced by parental school involvement, under conditions of a profound and prolonged socio-economic crisis. This particular configuration of variables was studied together for the first time, and was informed by economic, developmental and positive psychological considerations. Thus, the multitude of significant findings yielded span various scientific domains and can enhance socio-economic and psychological theory, research and practice. At the most applied level it can inspire positive developmental and educational interventions, and inform educational and social policy to enhance the well-being of emerging adults.

The first study hypothesis (a) regarding ISM was confirmed. Upward intergenerational educational mobility across three generations of Greeks was ascertained. Results from various analyses demonstrated that educational mobility was higher a generation back, i.e. parents were better educated than grandparents, with contemporary youths also achieving higher levels of education than their parents. Further, certain socio-economic differences between TEI and University students and graduates surfaced with respect to educational mobility over time. A generation back, when grandparents’ education was nonexistent or low, their offspring’s chances of entering University were virtually non-existent, while they had some chance of entering a TEI; at the same time, children of University educated grandparents entered TEIs more frequently, albeit at a low or moderate rate. Instead, they entered University at a higher rate. A generation later, fathers with non- or lower-educated parents had increased chances of studying at TEIs and, overwhelmingly high chances of studying at the University; the chances of fathers from highly educated family backgrounds rose to almost 97%. These findings can be explained by a tremendous education boost having taken place in Greece in the second half of the twentieth century, and largely continuing to date. This was facilitated by public policy decisions to level the inequalities created by two World Wars and a civil war in the last century through increased access to free education for more students, and the creation of many Universities and TEIs throughout the country starting at the 60s and continuing through the first two decades of the 2000s (Saiti & Prokopiadou, 2008). These decisions and practices led to many children of poorly and averagely educated parents attaining the highest education qualifications, thus gaining more advantageous positions in the workplace. Indeed, this can be corroborated by reports showing a leveling of educational attainment at the latter part of the twentieth century in Greece, as the particularly high enrollment in tertiary education reveals (Daouli et al., 2010; Galanaki & Sideridis, 2019).

The impact of these educational and socio-economic changes was reflected on the levels of well-being of young people (hypothesis b). Social mobility significantly and strongly affected emerging adults’ flourishing, but also their perceived quality of life. This finding is in line with previous research (Becker & Birkelbach, 2018). It confirms findings suggesting a strong link among mobility and mental health (Bjørnskov et al., 2013). As evidence of upward ISM was found in this study, the results confirm previous research that firmly linked socio-economic advancement to positive mental health (Bridger & Daly, 2020). In particular, upward ISM as indexed by socio-economic status and income was repeatedly found to positively affect quality of life, life and job satisfaction, social connectedness and mental health and happiness (Bridger & Daly, 2020; Li, 2016). The especially high association found in this study between educational mobility and flourishing, a measure created on the bases of positive psychological thinking, reinforces its basic position that mental health does not only constitute absence of psychopathology, but additionally requires presence of positive indicators of human functioning (Seligman & Csikszentmihalyi, 2014). Flourishing belongs to the latest measures of this tradition, and it is important to observe how closely it is linked to other measures of well-being, such as quality of life (with which is highly correlated), but also to educational mobility across generations. This latter finding is new and can inform the fields of developmental, positive, and social psychology, as well as economics, as ISM in this study was indexed at both the educational and income levels.

With respect to the mediational role of parental involvement on mobility and youth well-being (hypothesis c), it seems to be strongly associated to flourishing, a measure of internal self-evaluation of well-being; it does not relate to quality of life, a measure that reflects satisfaction with more diverse variables that may be perceived as not being exclusively under the control of the individual, including health and living conditions. As the economic theory of the distribution of income (Becker & Tomes, 1979) and the human capital theory (Becker, 1964) suggest, parental resource allocation in terms of human and non-human capital strongly affected emerging adults’ well-being, as well as their income mobility, through the level of parental school involvement. This is in line with previous studies that explored the role of parental control in adolescent development via the mediation of attributional style, self-regulation and emotional involvement (Schleider et al., 2014; Wenk et al., 1994). This finding may also signify that the interest parents take at their children’s schooling at a young age can equip them with sufficient skills that can potentially lead them to higher personal and interpersonal happiness in emerging adulthood. Only indirectly may these characteristics aid them in a course of a more generalized sense of quality of life. These links remain to be further explored. Finally, it should be noted that mobility was inversely related to parental involvement. This may be explained on the basis of the amount of time and energy that parents are required to spend on their better paid, but possibly more demanding jobs. In their efforts to achieve, their personal resources may be more depleted than if their career paths were less demanding, leaving them less time to be actively involved in their children’s schooling.

Regarding the effects of the severe crisis that the Greek society underwent in the 2010s, these appear to be pervasive for emerging adults’ flourishing, and especially high for their overall quality of life; thus, our fourth hypothesis (d) concerning the mediational role of the crisis between ISM and mental health was confirmed. According to the SEM model, the relationship between social mobility and crisis proved negative, signifying that upward mobility trajectories rendered participants better equipped to handle the effects of the crisis. Better paid jobs, resulting from higher education appeared to allow them more room to deal with the demands of their everyday lives. This in turn allowed participants and their families to maintain a certain level of quality of life, and also to flourish, as a lot of previous research evidence linking crises with well-being indicated (Kretsos, 2014; Papanastasiou & Papatheodorou, 2018). Young people who saw their personal and family financial situation significantly and continually deteriorating due to the socio-economic crisis also experienced the insecurity inherent in this, faced increased intergenerational tension, and a general decrease in quality of life (Gouglas, 2013). Evidence of youths overcoming the detrimental effects of the crisis emerged here, largely via the mediation of parental involvement in their children’s education. Once again, the adverse effects of the crisis were mitigated by the financial state of young people’s families, and their ability to meet the demands of their lives. As expected, those who were able to maintain a level of financial security exhibited higher flourishing and quality of life. Similarly, OECD data (2014) suggested that people with higher education were better able to avoid unemployment during the Great Depression of our times (2008–2014). The above results support several similar ones from studies in Greece and abroad (Bridger & Daly, 2020; Kokkevi et al., 2014; Leontopoulou, 2018; Motti-Stefanidi & Asendorpf, 2017). It should be noted that the crisis itself did not appear to affect the level of parental school involvement in the Greek society. This is congruent with the inherent features of both variables. Crises by their nature do not last long, and although in the case of Greece this crisis lasted for a good ten years, this was not enough to alter deep rooted characteristics and manifestations of perceived parental roles, such as caring for children’s education. Rather, the impact of the crisis was very strong on the quality of life of young people, as it affected their available income, their health and their relationships with others.

Throwing a developmental light on these results, important insights can be gained for the continuity of interpersonal, intrapersonal, as well as societal and economic processes from childhood to emerging adulthood. Intergenerational mobility, influenced by the educational attainments of grandparents, parents and youths, as well as the income levels of parents and young people affect flourishing and the quality of life of emerging adults. It does so both directly and indirectly, via the mediation of parental school involvement for flourishing, and the effects of the socio-economic crisis for both indicators. This is a novel finding, as to the best of our knowledge social mobility has not been hitherto examined with respect to a socio-economic crisis. It is, therefore, important to empirically confirm the hypothesized intergenerational influences that various mobility types exert on youth well-being and how these can be exacerbated under crisis. Also, the effects of ISM and intrapersonal and external variables were previously examined mainly for children and adolescents, but not for emerging adults. This age group comes with its own characteristics and challenges at different levels. On their way to adulthood, youths seem to take heterogeneous developmental paths, especially as far as optimism, resilience, and identity development in the face of adversity are concerned (Galanaki & Sideridis, 2019; Leontopoulou, 2018; Motti-Stefanidi & Asendorpf, 2017). Thus, transition to adulthood may follow more or less troubled pathways, depending on the social and financial status of youths and their families, and echoing these of their grandparents, according to results from the present study. Furthermore, the fact that University students and graduates tend to come from more affluent families, and, therefore, usually have higher prospects for upward ISM can influence their well-being and positive development, not to mention their chances of finding suitable employment, especially during economic crises. For the majority of youths the crisis clearly prolonged transition to adulthood, exacerbating young people’s financial dependence on their parents, their economic vulnerability, and further restricting their employment prospects and choices, thus rendering them “young precariat” and leading to a “Brain Drain”. It also led to a protracted period of identity exploration, limited adoption of adult roles, inability to exercise agency and a lower sense of commitment in a relationship (Galanaki & Sideridis, 2019; Kokkevi et al., 2014; Leontopoulou, 2018).

With respect to the final hypothesis (e) regarding the equivalency of the traditional pen-and-pencil and the online questionnaire completion, the results have been conclusive. No significant differences were found between the two modes of completion (with a single exception regarding young people’s perceptions of their parents’ hopeful life outlook). This finding becomes particularly insightful and useful today, when the multiple constraints imposed by the Covid-19 pandemic, including lockdowns in over 200 countries worldwide and the long-distance online education adopted by most countries to name but a few, also impact research practices. It is important for researchers to be adequately reassured as to the fact that the online surveys yield similar results to the pen-and-pencil ones.

### Limitations and Suggestions for Future Research

Despite this study’s many contributions to a number of scientific fields, it suffers certain limitations. Firstly, our non-probability sampling rendered participants mainly from North-Western Greece, who were predominantly female and studied in Higher Education institutions. In this country enrollment in tertiary education is particularly, high and parents tend to handsomely assist their children’s studies financially; hence, only about 20% of the sample worked, mainly part-time. Therefore, our results’ generalizability is limited. More research with other groups of young people is needed, including older students, more males, working part- and full-time, or in atypical employment, studying in other urban and rural areas, and originating from various cultural groups that exist in the country, and also NEETs. Secondly, the impact of the crisis was assessed by two custom made questions concerning participants’ perceived family financial situation and needs. Although these questions proved powerful predictors of youth well-being, more objective measures and structured questionnaires would benefit future studies. Thirdly, the scale used to measure parental school involvement was based on Epstein and Salinas' (1993) theoretical model of parental involvement, which identifies five basic obligations of parents and schools. Although these obligations may not cover all forms of possible parental school involvement, the parental behaviors canvassed in the study uncover some of its most important dimensions, which can facilitate flourishing in emerging adulthood. Fourthly, the results need to be further examined in connection with various developmental processes that take place in emerging adulthood, such as identity development, resilience building and other facets of mental and physical health. Finally, longitudinal designs would help to identify cause and effect in the multifaceted and dynamic pathways that lead to positive youth development.

## Conclusions

In conclusion, positive youth development, as demonstrated by heightened well-being levels can and does take place during emerging adulthood. It depends on one’s history of intergenerational social mobility, on intra- and interpersonal processes taking place since childhood, and on contextual factors, such as socio-economic conditions; the latter two mediate these complex relationships. The implications of these findings for the qualifications and resources that they can offer youths in terms of educational attainment, and employment and financial prospects are significant. They can be usefully employed to design targeted educational, mental health and other types of interventions in all life stages up to and including emerging adulthood. They can also aid theory development in different areas in psychology, economics and sociology, and guide future research. Importantly, they can inform social policy and health promotion to increase quality of life and flourishing for all. All the above can contribute to a larger, overarching goal, namely the reduction of inequality in modern society. The potential beneficial effects of increasing mobility across generations are far reaching, especially in an increasingly globalized economy such as ours, where new opportunities for wealth and income are emerging (HM Government, April 2011). Equalizing opportunities in education and occupation signifies an investment in a fair society, where these opportunities are open to everyone. Vitally and elegantly put, “A fair society is an open society. A society in which everyone is free to flourish and rise. Where birth is never destiny” (HM Government, 2011, p. 3).

## Data Availability

On request.

## Footnotes

1 Since this study was carried out, the two highest educational levels in Greece were merged, and henceforth constitute the highest educational level, i.e. University.

## Code availability

SPSS v. 22.
